# The place of VEGF inhibition in the current management of renal cell carcinoma

**DOI:** 10.1038/sj.bjc.6603025

**Published:** 2006-02-28

**Authors:** P Nathan, D Chao, C Brock, P Savage, M Harries, M Gore, T Eisen

**Affiliations:** 1 Mount Vernon Cancer Centre, Rickmansworth Road, Northwood, Middlesex, HA6 2RN, UK; 2 Royal Free Hospital, Pond Street, London, NW3 2QG, UK; 3 Charing Cross Hospital, Fulham Palace Road, London W6 8RF, UK; 4 Guy's and St. Thomas' Hospital, St. Thomas Street, London SE1 9RT, UK; 5 Royal Marsden Hospital, Fulham Road, London SW3 6JJ UK

**Keywords:** renal cell carcinoma, VEGF, vascular endothelial growth factor, sorafenib, sunitinib

## Abstract

Vascular endothelial growth factor (VEGF) is overexpressed in around 80% of patients with clear cell carcinoma of the kidney owing to the inactivation of von Hippel Lindau gene activity. VEGF stimulates angiogenesis and acts as an autocrine growth factor. A number of different agents are now available which target VEGF and its signalling pathways. A significant body of evidence has accumulated demonstrating that antagonism of VEGF and its downstream pathways is clinically useful in a significant proportion of patients with metastatic clear cell carcinoma of the kidney. Enough data is now available to recommend that patients with metastatic clear cell carcinoma of the kidney should at some point during the course of their disease be offered entry into a clinical trial enabling exposure to a targeted inhibitor of VEGF or its signalling pathways. Assuming early clinical trial data is substantiated by ongoing registration studies, efforts should be made to minimise the time taken between licensing and general availability of these active agents.

Systemic treatment options for renal cell carcinoma (RCC) have been extremely limited. Standard care for metastatic disease in the UK is with single-agent interferon-*α* which has a 15% response rate and increases 1-year survival from 31 to 43% (MRC Collaborators, 1999). High-dose interleukin-2 until recently was the only drug currently licensed by the FDA for the treatment of metastatic RCC and at best gives durable benefit in 5–10% of patients at the expense of significant toxicity (McDermott *et al*, 2005). Advances in the understanding of the molecular biology of the most common form of RCC, clear cell carcinoma, have shown that the von Hippel Lindau (VHL) gene is mutated in the majority of sporadic cases (Gnarra *et al*, 1994). Loss of VHL gene activity is known to deregulate hypoxia inducible factor (HIF) which in turn leads to an upregulation of expression of a number of genes including vascular endothelial growth factor (VEGF), platelet-derived growth factor, carbonic anhydrase IX, CXCR4 and tumour growth factor-*α* (Siemeister *et al*, 1996; Semenza, 2003). VEGF is known to be overexpressed in RCC (Nicol *et al*, 1997) and acts as a potent stimulator of angiogenesis.

## VASCULAR ENDOTHELIAL GROWTH FACTOR

The VEGF gene family consists of six related glycoproteins: VEGF-A, VEGF-B, VEGF-C, VEGF-D, VEGF-E and placenta growth factor. VEGF-A is commonly referred to as VEGF and was initially identified by its ability to increase vascular permeability. It is a dimeric glycoprotein which exists in four major isoforms (Tischer *et al*, 1991). Vascular endothelial growth factor is now known to have multiple effects, which promote endothelial cell activation, growth (Zachary and Gliki, 2001), longevity (Alon *et al*, 1995) invasion and migration (Zachary and Gliki, 2001).

Vascular endothelial growth factor signalling is mediated by binding to transmembrane tyrosine kinase receptors. VEGFR-1, VEGFR-2, VEGFR-3, Neuropilin-1 (NRP-1) and NRP-2 all bind members of the VEGF family and mediate downstream signalling. VEGFR-2 is thought to mediate most of the pro-angiogenic effects of VEGF (Millauer *et al*, 1993).

Vascular endothelial growth factor and its signalling pathways have been extensively reviewed (Ferrara *et al*, 2003; Hicklin and Ellis, 2005).

Two major anti-VEGF strategies namely, neutralising anti-VEGF antibody and small molecule multi-targeted kinase inhibitors, which have activity against VEGF and other signalling pathways, have been examined in recently reported clinical trials to date (Table 1).

### Bevacizumab

Bevacizumab (Avastin) is a humanised neutralising anti-VEGF antibody (Presta *et al*, 1997) which binds to all biologically active isoforms of VEGF. The original murine antibody, A.4.6.1, had been shown to have anti-tumour activity in pre-clinical models. In phase I studies, bevacizumab was shown to have a half-life of approximately 21 days (Gordon *et al*, 2001). Most patients tolerated the antibody well although haemorrhage, thrombosis, bowel perforation, hypertension and proteinuria have all been reported (Gordon *et al*, 2001; Yang *et al*, 2003; Hurwitz *et al*, 2004).

A prospective randomised double-blind three-arm phase II study of low (3 mg kg^−1^) and high (10 mg kg^−1^) dose bevacizumab given 2-weekly *vs* placebo in metastatic RCC was the first controlled study demonstrating clinical activity of an anti-VEGF approach in RCC (Yang *et al*, 2003). A total of 116 patients with metastatic clear cell RCC were randomised with 37–40 patients on each arm. All patients had documented evidence of progression at study entry and almost all had received previous cytokine therapy. Of 116 patients, 91 were ECOG performance status 0 with the remainder being ECOG PS 1. The three treatment arms were well balanced in terms of MSKCC prognostic indicators (Motzer *et al*, 1999) (performance status, anaemia, hypercalcaemia, prior nephrectomy). Primary objectives were time to progression (TTP) and overall response rate. Survival was a secondary end point as patients who progressed on the placebo arm were allowed to crossover to low-dose bevacizumab or entry into a study of bevacizumab in combination with thalidomide. The drug was well tolerated with 21% of patients in the high-dose arm experiencing grade 3 hypertension and 64% in the same treatment arm experiencing asymptomatic proteinuria with no reduction in renal function. Toxicities reversed upon stopping treatment. No treatment-related deaths were seen.

The study was stopped early by the data monitoring committee according to O'Brien-Fleming rules. A highly significant improvement in TTP (HR: 2.55, *P*<0.001) was seen in the high-dose arm compared to placebo. Median TTP of the high-dose arm was 4.8 months compared to 2.5 months in the placebo arm and the overall response rate in the high-dose arm was 10%. There was a non-statistically significant improvement in TTP of the low-dose arm compared to placebo. No significant survival advantage was seen although the trial was not powered for this end point.

A phase III registration study of bevacizumab *vs* interferon-*α* in the first line treatment of metastatic RCC has recently been performed and results are awaited.

Bevacizumab is also being evaluated in combination with other targeted therapies. The epidermal growth factor receptor (EGFR) is commonly expressed in RCC (Langner *et al*, 2004). Single-agent gefitinib (Iressa), a targeted EGFR-tyrosine kinase inhibitor, showed little evidence of single-agent activity in RCC (Dawson *et al*, 2004). However, in pre-clinical models, downregulation of EGFR resulted in downregulated VEGF expression (Riedel *et al*, 2002). The combination of bevacizumab and erlotinib (Tarceva), another EGFR tyrosine kinase inhibitor demonstrated a 25% response rate with a further 61% of patients having stable disease at 8 weeks in a 63-patient phase II study (Hainsworth *et al*, 2005). Median progression-free survival was 11 months and at 15 months of follow-up, median overall survival had not been reached. However, a recent announcement from Genetech has indicated that in the randomised phase II trial of bevacizumab *vs* bevacizumab plus erlotinib there were no advantages to the combination (www.gene.com, press release 18 October 2005).

The efficacy of combination-targeted treatment will only be fully described in randomised studies but early data is interesting enough to warrant further combination studies. Currently, phase I/II studies of bevacizumab in combination with sorafenib (see below), CCI-779 (an mTOR inhibitor – Temsirolimus, Wyeth), and Interleukin-2 are ongoing.

### Small-molecule targeted therapies

A number of small-molecule multi-targeted kinase inhibitors are under investigation. They inhibit signalling mediated by the type 2 VEGF receptor as well as many other signalling pathways. All of these orally active drugs have predictable manageable toxicities and appear well tolerated.

#### Sorafenib

Sorafenib (BAY43-9006) is a bi-aryl urea and was originally developed as a raf kinase inhibitor. It has IC50s in the nanomolar range against VEGFR-2, VEGFR-3, PDGFR, flt-3, c-kit as well as craf and braf kinases (Wilhelm *et al*, 2004).

Dose-limiting toxicity in phase I studies was diarrhoea and fatigue at 800 mg b.d. and skin toxicity at 600 mg b.d. (Awada, 2005; Clark *et al*, 2005; Moore *et al*, 2005; Strumberg *et al*, 2005).

The recommended phase II dose of 400 mg b.d. was examined in a randomised discontinuation phase II study (Ratain *et al*, 2005). In this novel trial design, all patients received the standard dose of 400 mg b.d. After an evaluation at 12 weeks, patients who had a >25% response continued on active drug whereas patients with progressive disease (>25% tumour growth) were taken off study. Those with stable disease were randomised to receive either study drug or placebo. At progression, those receiving placebo were allowed to crossover back to active drug. This trial design has the advantage of accurately discriminating the proportion of patients who have disease stabilisation due to drug activity rather than to the biology of their disease.

Of the 202 metastatic RCC patients entering the study, 144 (71%) had tumour shrinkage or disease stabilisation at 12 weeks. Sixty-five patients entered the randomised phase and after a further 12-week period 16 (50%) of patients on sorafenib were progression free compared with six (18%) of patients on the placebo arm (*P*=0.0077). The median progression-free survival (PFS) from randomisation was 24 weeks for sorafenib compared with 6 weeks for the placebo arm (*P*=0.0087).

Preliminary data from a randomised placebo-controlled phase III trial in the second-line treatment of 903 patients with metastatic RCC has been reported this year (Escudier *et al*, 2005). All patients had received one prior systemic treatment in the 8 months before study entry. Only patients with clear cell carcinoma in the ‘Motzer’ good and intermediate prognosis groups were recruited. Treatment and placebo arms were well balanced in terms of prognostic factors and 57% of patients on each arm had greater than two metastatic sites of disease. Over 80% of patients had received cytokine-based therapy as first-line treatment and over 90% of patients had a previous nephrectomy.

The incidence of grade 3/4 toxicity was low. Six percent of patients experienced a grade 3-4 hand–foot syndrome and 4% hypertension. Haematological and biochemical grade 3/4 toxicities were rare with lymphopenia, hypophosphataemia and elevated lipase marginally more common in the treatment arm. The most common grade 1/2 adverse event was diarrhoea (43% sorafenib *vs* 13% placebo). Twelve percent of patients experienced a dose reduction, mainly due to hand–foot syndrome or diarrhoea. Twenty percent of sorafenib patients had a dose interruption (5% placebo). There was no significant difference between the sorafenib and placebo arms (10 *vs* 8%) in terms of discontinuation of drug.

At the planned interim analysis after 220 events, a 10% partial response rate and 74% disease stabilisation rate was seen on the sorafenib arm compared with 2 and 53%, respectively on the placebo arm. The median PFS was 5.5 *vs* 2.8 months (HR: 0.51). The median overall survival of the placebo arm was 14.7 months and at the time of analysis had not yet been reached in the sorafenib arm (HR: 0.72, *P*=0.018). All subsets (age, prognostic group, sites of metastasis, previous cytokine treatment) appeared to derive equal benefit (Escudier presentation, ECCO 13, Paris 2005).

#### Sunitinib

Sunitinib (SU011248, Pfizer) is an orally active multi-targeted receptor tyrosine kinase inhibitor with activity against VEGFR, PDGFR, KIT, and FLT3 kinases. It showed significant pre-clinical activity against a variety of xenograft models (Mendel *et al*, 2003).

Two phase II studies have examined the activity of sunitinib in clear cell metastatic RCC in a total of 169 patients. Entry criteria for both studies included failure of previous cytokine therapy, adequate end-organ function and good performance status. Clear cell histology was stipulated in the second larger study (*n*=106), whereas 87% of patients in the earlier study (*n*=63) had clear cell histology. Nephrectomy was mandatory in the second study. Data from both studies has been combined in a meta-analysis and an update recently reported (Hudes, G.R., ECCO, Paris 2005).

Patients were treated with sunitinib 50 mg daily for a 4-week on, 2-week off 6-week cycle. The drug is well tolerated. Fatigue is the most common grade 3 toxicity (11% of patients) with stomatitis and hand–foot syndrome also noted. Neutropenia, increases in serum amylase and lipase were also seen. Twenty-seven percent of patients across both studies were dose reduced to 37.5 mg, 6% of patients were dose reduced to 25 mg. FACIT fatigue scores demonstrated fatigue during the 4-week on treatment period which recovered during the 2-week off period. Overall however, levels of fatigue were not significantly different from the general non-anaemic cancer population.

Pooled analysis of the two studies (*n*=168) showed a 42% overall response rate with a further 24% of patients with stable disease for longer than 3 months. Median PFS was 8.2 months. The PFS for patients who had true RECIST responses was significantly longer than patients who had stable disease as their best response (14.8 *vs* 7.9 months). Median overall survival for the first study was 16.4 months and at the time of reporting had not yet been reached for the second study.

A phase III study comparing sunitinib with interferon in the first-line treatment of metastatic RCC has recently been performed and results are awaited.

#### AG-013736

AG-013736 (Pfizer), another multi-target kinase inhibitor with nanomolar IC50s against all three VEGF receptors and PDGF-R*β* has been examined in a phase II study of 52 metastatic RCC patients (Rini *et al*, 2005). Drug was given orally at 5 mg b.d. Patients were of good performance status, had failed one previous cytokine-based therapy and any hypertension had to be well controlled as a pre-requisite for study entry. Grade 3/4 toxicity was hypertension (15%), diarrhoea (8%) and fatigue (8%). Forty-six percent of patients had a partial response with a further 38% of patients having some shrinkage in the size of their disease. Only 14% of patients had no response. At 12–18 months of follow-up, median TTP had not yet been reached.

The drug will be examined in disease that has become refractory to other targeted kinase inhibitors.

### Surrogate markers of activity

As clinical experience grows with targeted kinase inhibitors, surrogate markers are being identified that reflect exposure to drug. Both sorafenib and sunitinib induce an increase in circulating VEGF levels and a decrease in soluble VEGFR levels (Escudier *et al*, 2005; Norden-Zfoni *et al*, 2005). Changes in monocyte levels and circulating endothelial cells have also been noted with sunitinib (Norden-Zfoni *et al*, 2005). No surrogate marker has yet been shown to conclusively correlate with anti-tumour effect but the hope is that patients who are likely to derive clinical benefit will ultimately be identified early using surrogate markers of response.

## CONCLUSION AND RECOMMENDATIONS

Mature phase III data from the lead compounds is awaited. However, the available phase II and phase III data shows significant activity in both progression-free survival and clinical benefit (CR+PR+SD). Early indications are that there will be a significant effect upon overall survival. Given that this is a disease with few active treatment options for the majority of patients, there is now a significant body of data demonstrating that this class of agents has useful clinical activity.

We therefore believe that all metastatic clear cell RCC patients should at some point during their treatment have the opportunity to be exposed to a VEGF inhibitor. Patients who are of reasonable performance status and who do not have any specific contraindications for anti-VEGF treatment should therefore be referred to a centre that has access to an appropriate agent within the context of a clinical trial. Pharmaceutical companies should be encouraged to expand their planned extended-use programmes to enable patients to access these active drugs pre-licensing. Commissioners should be made aware of the fact that once licensed, these drugs should be made available for a patient population which up until now has had no adequate therapeutic options.

## Figures and Tables

**Table 1:** Activity of anti-VEGF agents in metastatic renal cell carcinoma trials

	**No**	**ORR (%)**	**CR+PR+SD (%)**	**PFS /TTP (months)**
*Sunitinib (Phase II)*				
Trial 1	63	40	67	8.2 (pooled analysis both studies)
Trial 2	105	44	67	
				
Sorafenib (Phase III)	451	10	74	5.5
AG-013736 (Phase II)	52	46	86	Not reached
Bevacizumab (Phase II)	39	10		4.8
